# A171 A MISSENSE MUTATION IN FCGBP, A STRUCTURAL COMPONENT OF MUC2 MUCUS, ALTERS THE GLYCOMICS PROFILE AND FUNCTION OF COLONIC MUCUS

**DOI:** 10.1093/jcag/gwad061.171

**Published:** 2024-02-14

**Authors:** H Gorman, F Moreau, W Zandberg, K Bergstrom, K Chadee

**Affiliations:** University of Calgary, Calgary, AB, Canada; University of Calgary, Calgary, AB, Canada; University of British Columbia Okanagan Campus, Kelowna, AB, Canada; University of British Columbia Okanagan Campus, Kelowna, AB, Canada; University of Calgary, Calgary, AB, Canada

## Abstract

**Background:**

MUC2 mucin produced by colonic goblet cells, form a mucus bilayer that provides a physical barrier between potential pathogens in the lumen and the underlying epithelial cells. Mucus is thus the first line of innate host defense in the gastrointestinal (GI) tract. Many GI diseases including inflammatory bowel disease and colon cancer affect the glycosylation of mucus. Goblet cells produce a variety of proteins that are associated with the mucus layer. Of these proteins, FCGBP is of significant interest due to its structural similarities to MUC2 mucin with unknown functions. In this study, we investigated if a missense mutation in FCGBP altered the glycosylation of goblet cell MUC2 and affected its barrier functions.

**Aims:**

Aims:

1) To determine mechanistically how FCGBP impeded the structural integrity of the mucus layer

2) To quantify MUC2 glycoprotein modifications in the altered mucus layer

**Methods:**

To investigate whether FCGBP impaired mucus barrier functions, two cell types were investigated: wildtype LS174T (WT) MUC2 mucus-producing goblet cells and LS174T cells with a missense mutation in FCGBP (FCGBP-*Mut*). To determine if FCGBP-*Mut* led to loss in barrier function in the mucus layer, the penetration of 0.2, 1, and 2 μm fluorescent beads (to mimic bacteria) through the mucus layer were quantified. To determine if the differences in penetrability were caused by differences in MUC2 glycosylation in the goblet cell lines, sensitive glycomic analyses were performed by high-performance liquid chromatography-mass spectrometry (HPLC-MS) and capillary electrophoresis with laser-induced fluorescence detection (CE-LIF). Both cells and purified MUC2 mucin granules were analyzed and confirmed by lectin binding assays. To enumerate differences in the glycomics profiles, RT-PCR was performed on over 30 human glycosyltransferases.

**Results:**

FCGBP-*Mut* cells exhibited an altered glycomics profile with a significant increase in sialylated/fucosylated glycans as quantified by HPLC-MS and an increase in sialyl- and fructosyltransferase mRNA expression. In contrast to WT goblet cells, FCGBP-*Mut* cells exhibited significant loss in MUC2 mucus barrier function as quantified by fluorescent beads penetration through the mucus layer temporally. The altered glycosylation in FCGBP-*Mut* cells triggered increased metabolic stress and ROS production that enhanced susceptibility to *Salmonella enterica* binding, invasion and cell death.

**Conclusions:**

This study demonstrates that a single missense mutation in FCGBP altered the penetrability of the mucus layer associated with an increase in sialylated/fucosylated glycans, a signature hallmark of numerous colonic diseases. These findings underscore the importance of FCGBP in providing structural integrity of the mucus layer and homeostatic maintenance of goblet cell glycosylation profiles.

**Funding Agencies:**

CIHR

